# Studies of Raman-Scattered Technology on S-Shaped Dinaphtho[2,1-b:2′,1′-f]thieno[3,2-b]thiophene-10 (S-DNTT-10)

**DOI:** 10.3390/ma18102389

**Published:** 2025-05-20

**Authors:** Haobing Wang, Olivier Simonetti, Oumaima Et-Thakafy, Nicolas Bercu, Florence Etienne, Sylvain Potiron, Pierre-Michel Adam, Louis Giraudet

**Affiliations:** 1 Light, Nanomaterials, Nanotechnologies (L2n) Laboratory, CNRS UMR7076, University of Technology of Troyes, 12 Rue Marie Curie, 10004 Troyes, France; pierre_michel.adam@utt.fr; 2 Université de Reims Champagne Ardenne, L2n-URCA, 51100 Reims, France; ouma.takafi@gmail.com (O.E.-T.); nicolas.bercu@univ-reims.fr (N.B.); florence.etienne@univ-reims.fr (F.E.); sylvain.potiron@univ-reims.fr (S.P.)

**Keywords:** S-DNTT-10, Raman scattered, DFT simulation, SERS effect

## Abstract

S-shaped dinaphtho[2,1-b:2′,1′-f]thieno[3,2-b]thiophene (S-DNTT) molecules have shown promise for applications in organic electronic devices, though their molecular characteristics are not fully understood yet. In this study, we first revealed the material characteristics of S-DNTT-10 by vibrational dynamics using Raman spectroscopy and density functional theory (DFT) simulations, employing the B3LYP functional method and the 6-311G (d, p) basis set. The molecular vibrations identified included C–H bending in alkyl chains and the deformation of S-shaped thiophene rings. In addition, surface-enhanced Raman scattering (SERS) with 785 nm incident light was applied to thermally deposited 25 nm S-DNTT-10 thin films with gold (Au) nanostructures. It showed enhanced Raman signals from the lower S-DNTT-10 layers. The findings significantly contribute to the knowledge of S-DNTT-10 molecular properties and also contribute insights into using this material into organic electronic devices in the future.

## 1. Introduction

Due to the unique advantages of organic semiconductor (OSC) materials, making organic electronic devices, such as organic thin-film transistors (OTFTs), comes with advantages, such as high flexibility, large-area fabrication compatibility, and lower temperature manufacturing, compared to inorganic counterparts [1,2,3,4,5,6,7]. These characteristics render OTFTs highly promising for integration into commercial organic electronics. To date, OTFTs have been successfully used in various applications [8,9,10,11,12,13].

**Figure 1:**
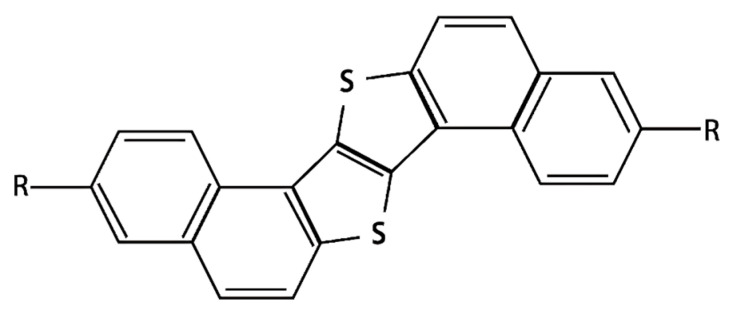
S-shaped dinaphtho[2,1-b:2′,1′-f]thieno[3,2-b]thiophene-10, S-DNTT-10 (R = −CH_3_(CH_2_)_9_).

In the field of organic thin-film transistors (OTFTs), organic semiconductors (OSCs) play a critical role in determining device performance [14,15,16,17]. Historically, OTFTs have employed OSC layers with relatively simple molecular structures, such as acenes, heteroacenes, and their derivatives [18,19]. However, OTFT development has advanced significantly with the introduction of OSCs featuring larger and more complex molecular architectures [20,21,22,23,24,25,26,27,28,29]. A notable example is the S-shaped dinaphtho[2,1-b:2′,1′-f]thieno[3,2-b]thiophene (S-DNTT) family, particularly S-DNTT-10, as shown in Figure 1, which is characterized by a distinctive S-shaped thiophene core and alkyl chains composed of ten carbon atoms [30]. This molecular structure facilitates efficient charge transport, enabling charge carrier mobility of several cm^2^·V^−1^·s^−1^ in thermally deposited OTFTs. Moreover, its improved stability under ambient conditions highlights its suitability for organic electronic applications [30]. Despite its promising performance and growing application prospects, the molecular characteristics of S-DNTT-10 are still not fully understood. Particularly, for S-DNTT-10, as one novel OSC material, the corresponding Raman spectra has not been investigated yet. Given its considerable potential in organic electronic devices, further investigations are essential to achieve a comprehensive understanding of its molecular properties.

Raman scattering [31], including surface-enhanced Raman scattering (SERS) [32], is a well-established, non-destructive technique for probing molecular characteristics that has been largely used on OSC materials. Building upon previous preliminary research [30], this study aims to investigate the molecular vibrational spectra of S-DNTT-10 using Raman scattering. Initially, the Raman spectra of S-DNTT-10 is analyzed by comparing experimentally obtained non-resonant Raman spectra with simulated spectra through Density Functional Theory (DFT), providing detailed insights into its molecular vibrational modes. These results fill a gap in knowledge regarding the Raman spectral characteristics and vibrational mode of S-DNTT-10. Subsequently, the weak Raman signal of a 25 nm thick S-DNTT-10 thin film was enhanced by thermally depositing gold (Au) films measuring several nanometers thick, resulting in the formation of Au nanostructures. This enhancement is primarily attributed to SERS. The findings suggest that the degree of Raman signal enhancement depends on both the thickness of the deposited Au films and the wavelength of the incident light.

## 2. Materials and Methods

### 2.1. Fabrication of S-DNTT-10 Thin Films

All S-DNTT-10 thin films were made by the PVD (Physical Vapored Deposition; Vinci Technologies, Nanterre, France) method, on heavily doped p-type Si (100) wafers (0.02 Ω/cm) covered with a 99 nm SiO_2_ dielectric layer (Fraunhofer, München, Germany). Then, the Si/SiO_2_ substrate was cleaned via ultrasonication in acetone, isopropanol, and Milli-Q water for removing contaminants or impurities that could possibly have degraded the quality of deposited S-DNTT-10. Subsequently, the substrates were left stationary in a chemically deposited chamber for approximately 16 hours at room temperature to form approximately a few nanometers worth of self-assembled monolayer (SAM) layers named HMDS (1,1,1,3,3,3-Hexamethyldisilazane; Sigma-Aldrich, St. Louis, MO, USA) with the purpose of adjusting the deposited organic molecular arrangement. Then, the S-DNTT-10 bulk material (TCI EUROPE N.V., Zwijndrecht, Belgium) was deposited onto the Si/SiO_2_ substrate with the surface modification layer, HMDS, at the defined deposited rate of 0.1–0.3 Å·s^−1^ under a vacuum environment (~10^−7^ Pa) and at room temperature.

### 2.2. Fabrication of Gold (Au) Nanostructures

The Au nanoparticles in this study were thermally deposited in the form of nano-layers on the S-DNTT-10 thin films at a rate of 1.0 Å·s^−1^ under room temperature in a vacuum environment (~10^−7^ Pa).

### 2.3. Computational and Test Details

Theoretical simulations of the S-DNTT-10 monomer are executed by density functional theory (DFT) simulations for calculating ground-state energy and non-resonant Raman spectra on an S-DNTT-10 monomer. The DFT simulations were performed by means of the **Gaussian 16** package using Beche’ nonlocal three-parameter exchange and the correlated functional with the Lee–Yang–Parr correlation (**B3LYP**) functional method and the **6-311G+ (d, p)** basis set (‘+’ as one diffusion function to strengthen the quality of the simulated results). The details about modeling S-DNTT-10 molecular structure are shown in Figure S1, and a summary of the Gaussian calculation parameters including the image of optimized molecular structure with ground state energy is, respectively, provided in Figure S2a,b.

The Raman spectroscopy is from Horiba Company, Tulln, Austria. And the atomic force microscopy (AFM) is from Bruker, Billerica, Massachusetts.

## 3. Results and Discussion

### 3.1. Theoretical Calculation and Measured Raman Spectra

**Figure 2:**
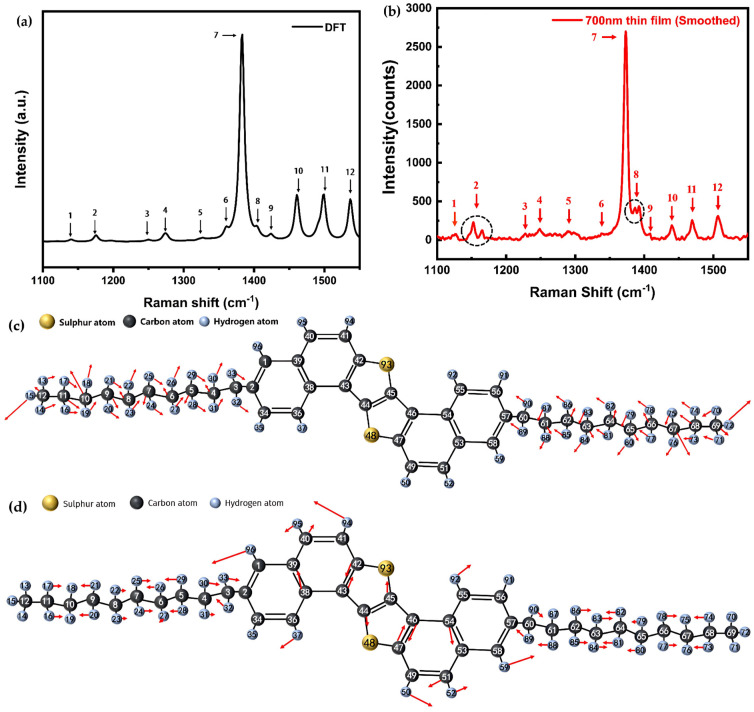
(**a**) The DFT simulation of non-resonance Raman spectrum of S-DNTT-10 in the range of 1100–1550 cm^−1^, the overall spectrum in Figure S2, (**b**) the measured non-resonance Raman spectrum of 700 nm S-DNTT-10 thin film in 1100–1550 cm^−1^ range. (The excitation wavelength = 532 nm, illumination laser power = 6.22 mW, objective lens 50×, N.A. = 0.5; exposure time = 30 s, accumulation times = 3, the used grating of 1800 grooves/mm) (**c**) the vibrational mode 1, and (**d**) the vibrational mode 7.

To investigate the Raman spectrum of S-DNTT-10, Figure 2a presents the simulated non-resonant Raman spectrum by DFT simulation in the 1100–1550 cm^−1^ range, which displays twelve distinct peaks labeled 1–12. And Raman spectroscopy was employed to analyze the experimental Raman spectrum of S-DNTT-10 under 532 nm incident light with an output power of approximately 6.22 mW. Figure 2b presents the Raman spectrum obtained from a 700 nm thick S-DNTT-10 film. According to the DFT simulation, these peaks correspond to specific vibrational modes of the S-DNTT-10 molecule, each associated with unique atomic displacement patterns. For example, modes 1 and 7, illustrated in Figure 2c and Figure 2d, respectively, demonstrate the diversity of vibrational behavior. Red arrows indicate atomic displacement directions, with arrow lengths proportional to displacement magnitude. Mode 1 is characterized by C–H bending in the alkyl chains, coupled with deformation of the thiophene rings. In contrast, mode 7 involves significant deformation of the aromatic and thiophene rings, along with C–H bond bending. Detailed descriptions of these modes are summarized in Table 1, offering insights into the vibrational characteristics of S-DNTT-10.

The comparison between the theoretical and experimental Raman spectra of S-DNTT-10 revealed that, although certain experimental peaks align closely with the theoretical predictions, notable discrepancies are evident (Figure 2a). For instance, pronounced peak splitting in vibrational modes 2 and 8 exhibited markedly from theoretical predictions. These discrepancies are most likely attributable to Davydov splitting [33], which arises due to variations in intermolecular interactions among adjacent molecules. In fact, Davydov splitting, known as correlation field splitting, refers to splitting in the electronic or vibrational spectra of crystals due to interactions among multiple equivalent molecular entities within a single unit cell. A splitting between 1158 cm^−1^ (m_0_ band) and 1155 cm^−1^ (m_1_ band) was previously attributed to Davydov splitting by Jentzsch et al. [34] and confirmed by He et al. [35]. He et al. [35] highlighted that Davydov splitting primarily arises from molecular packing and polycrystallinity, and defects and grain boundaries. The first factor is attributed to the imperfect molecular stacking structures that are found in polycrystalline and polycrystalline films, in contrast to single crystals, causing variations in intermolecular interactions. The strength of intermolecular interactions and the stacking configuration directly influence the energy distribution of molecular vibrational modes, potentially giving rise to Davydov splitting. The second factor involves defects and grain boundaries, which reduce molecular orbital overlap due to structural imperfections, thereby altering excited-state energy transfer pathways and influencing the split phenomenon. This effect is particularly pronounced in polycrystalline films, where defects and irregular molecular arrangements may further exacerbate the splitting. Consequently, based on the above analysis, it is plausible that the molecular arrangements became increasingly disordered during the deposition process of the S-DNTT-10 thin film, contrasting with the initially well-ordered molecular layers formed on the SiO_2_ substrate. The disparity in molecular orientation is likely the main contribution to the observed peak splits in mode 2 and mode 8.

Figure 2b presents the non-resonant Raman spectrum of S-DNTT-10 and demonstrates how it closely aligns with the simulated spectrum shown in Figure 2a. The simulations effectively reveal the vibrational characteristics of the gas-phase S-DNTT-10 monomer. The spectral features are similar to those observed in other OSCs, such as DNTT [17], particularly in the intensity variations across distinct vibrational modes. According to classical Raman theory, the Raman scattering intensity is proportional to changes in molecular polarizability. Consequently, variations in peak intensity arise from the fluctuations in polarizability associated with specific molecular vibrations. Density functional theory (DFT) simulations indicate that these modes primarily involve bending and deformation of molecular bonds. Dynamic structural variations during vibrations directly modulate the Raman scattering cross-section. For instance, the peak at ~1373.03 cm^−1^ (mode 7) exhibits the highest intensity, while mode 1 (~1150 cm^−1^) shows comparatively lower intensity. According to the analysis summarized in Table 1, mode 7 involves great C–C stretching within the thiophene rings, coupled with C–H bending. In contrast, mode 1 primarily comprises C–H bending in the alkyl chains and makes minor contributions via bond stretching.

Despite Figure 2b displaying the non-resonant Raman spectrum of S-DNTT-10, and the simulations in Figure 2a providing theoretical insights into the corresponding molecular characteristics, certain discrepancies were identified between the experimental and simulated spectra, which are similar to previously reported deviations observed in DNTT and pentacene prior to the application of scaling factors [17,36]. These differences are likely attributable to the inherent discrepancy between densely packed molecules in experimental solid-phase thin films and the isolated gas-phase molecular conditions that are typically assumed in DFT simulations [17]. Compared to the isolated molecules, densely packed molecular arrangements inherently involve modified intermolecular interactions, thus altering electronic distributions and molecular polarizabilities. Consequently, the experimentally observed peak positions and intensities may deviate from theoretical predictions.

These spectral variations primarily originate from intermolecular interactions within the thin film structure, significantly influencing Raman spectral characteristics. Although DFT simulations provide a valuable theoretical framework for understanding molecular vibrations and their associated spectroscopic properties, they cannot fully capture the complexities arising from intermolecular interactions inherent in condensed-phase systems, such as thin films. This limitation underscores the necessity of integrating theoretical predictions with experimental observations to achieve a comprehensive characterization of the Raman spectral properties of OSCs, thus bridging the gap between idealized theoretical models and experimentally observed complex phenomena.

### 3.2. The SERS Effect on S-DNTT-10 Thin Film

**Figure 3:**
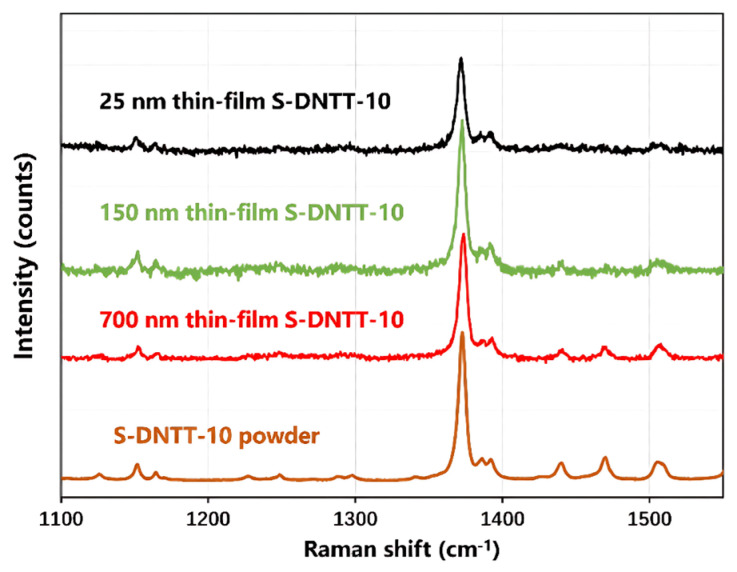
The non-resonant Raman spectra of the S-DNTT-10 thin film with different thicknesses using a 532 nm laser.

In the previous section, the Raman spectrum of a 700 nm thick S-DNTT-10 film was compared with the DFT-simulated spectrum of an isolated S-DNTT-10 molecule. Further investigation, as shown in Figure 3, revealed a pronounced dependence of Raman scattering intensity on the thickness of S-DNTT-10 films. Notably, as film thickness increases, the experimental Raman spectra progressively approach the spectral characteristics of powdered S-DNTT-10, exhibiting enhanced spectral clarity and detail. In contrast, much thinner films—for instance, a 25 nm S-DNTT-10 film—exhibit fewer distinguishable vibrational modes, with signals largely obscured by background noise. This observation indicates that thinner films result in a reduced detectability of specific Raman modes, highlighting the pivotal influence of film thickness on Raman signal quality and clarity. Consequently, thicker films produce stronger and more distinct Raman signals, whereas thinner films yield weaker signals that are more susceptible to noise. These findings underscore the critical role of film thickness in determining Raman spectral quality and the detectability of vibrational modes in S-DNTT-10.

In practical applications, particularly organic thin-film transistors (OTFTs), the thickness of the organic semiconductor (OSC) film significantly influences access resistance, thus influencing overall device performance. Notably, thinner films—typically on the order of tens of nanometers—can effectively lower access resistance and enhance performance, particularly in top-contact bottom-gate (TCBG) OTFT configurations [37,38,39]. However, as shown in Figure 3, reducing film thickness adversely affects the Raman signal intensity of S-DNTT-10 films, as evidenced by the comparative spectra of 25 nm and 700 nm films. This limitation presents analytical challenges for conventional Raman spectroscopy in the characterization of S-DNTT-10 with organic electronics.

To address this limitation, surface-enhanced Raman scattering (SERS) provides an effective approach for amplifying Raman signal intensity [40,41,42,43,44]. Inducing SERS enables the enhancement of the detectability of Raman scattered signals from thin films, facilitating detailed investigations of materials with inherently weak Raman scattering, low molecular concentrations, or ultrathin film architectures. SERS thus holds substantial promise for extracting rich spectroscopic information from S-DNTT-10 films at markedly reduced thicknesses and is expected to provide deeper insights into the molecular properties that are relevant to OTFT operation. Reference [45] demonstrates the effectiveness of SERS in significantly enhancing the Raman signals of pentacene in OTFT configurations, achieved by depositing an Au thin film onto pre-deposited electrode regions and subsequently characterizing these regions using a 785 nm excitation laser.

Previous studies have demonstrated that optimizing the thicknesses of gold electrodes can enable effective surface-enhanced Raman scattering (SERS) without adversely influencing device performance [45]. Considering the commercial potential of S-DNTT-10 in organic electronics and the feasibility of Raman-based characterization near electrode interfaces, this study proposes the thermal evaporation of gold (Au) thin films to construct dual-functional nanostructures. These structures simultaneously serve as electrodes S-DNTT-10-based OTFTs and enable SERS.

To optimize the SERS effect in OTFTs incorporating S-DNTT-10, Au thin films were thermally deposited onto regions covered with approximately 25 nm thick S-DNTT-10 films. The primary objective was to identify the appropriate Au film thickness for maximizing the SERS effect. To this end, Au films were systematically deposited with thicknesses of 3, 5, 10, and 20 nm at a deposition rate of 1.0 Å/s under high vacuum conditions. Furthermore, three distinct laser excitation wavelengths (532 nm, 633 nm, and 785 nm) were employed to comparatively assess their efficiencies in enhancing Raman scattering.

**Figure 4:**
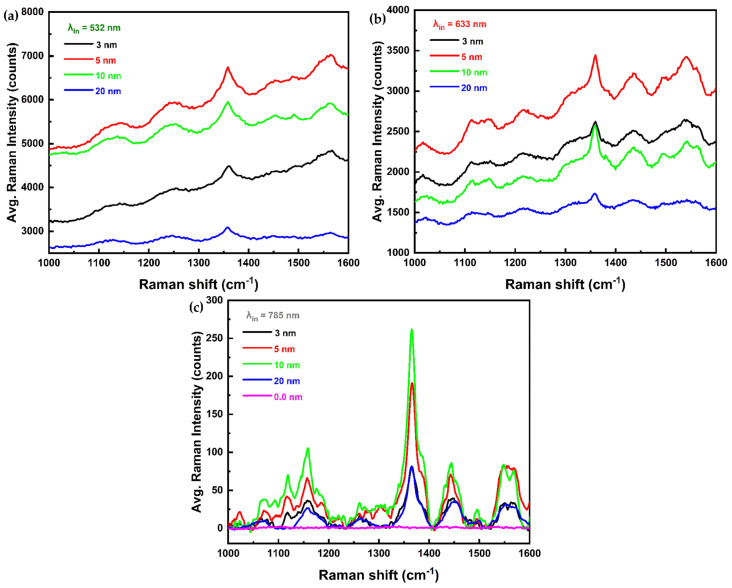
The SERS spectra for S-DNTT-10 molecules depositing by different thickness of Au thin films under lasers of (**a**) 532 nm, output power = 3.11 mW, exposure time = 20 s, accumulation times = 2, grating = 300 grooves/mm, (**b**) 633 nm, output power = 0.605 mW, exposure time = 17 s, accumulation times = 2, grating = 300 grooves/mm and (**c**) 785 nm with baseline-treatment, output power = 2.1 mW, exposure time = 14 s, accumulation times = 2, grating = 300 grooves/mm.

The “0 nm” label in Figure 4 refers to the Raman signal obtained from a pristine 25 nm thick S-DNTT-10 film. As shown in Figure 4a, the Raman spectrum acquired using 532 nm excitation displays pronounced background noise, with intensity increasing at higher Raman shifts. It indicates that 532 nm excitation is unsuitable for effective SERS with Au-based nanostructures. This conclusion is further supported by reference [45], which confirms the inadequacy of 532 nm excitation for SERS involving Au nanostructures. Similarly, the Raman spectrum obtained at 633 nm excitation (Figure 4b) exhibited extensive background noise, similar to that at 532 nm. In contrast, the Raman spectrum obtained using 785 nm excitation (Figure S3) clearly shows a lower background noise. As a result of this, subsequent research has focused specifically on the 785 nm excitation wavelength.

As shown in Figure 4c, an effective Raman signal was obtained under 785 nm excitation following smoothing and baseline correction. The result exhibited a noticeable enhancement in Raman signal intensity compared to the pristine S-DNTT-10, as indicated by the purple trace. This enhancement, attributed to the presence of metallic nanostructures, is consistent with previous studies [43,44] that reported the enhancement of cannabinol’s (CBN) Raman signal intensity using Au nanorods arrays and pentacene’s Raman signal intensity using Ag nanoparticles, respectively. Furthermore, the wavelength-dependent Raman response illustrating in Figure 4 aligns with previous reports [45], which demonstrated variations in Raman intensity across different excitation wavelengths for Au-film/pentacene structures. To ensure the reliability of the results, multiple 25 nm thick S-DNTT-10 films with identical Au thicknesses were fabricated, and Raman spectra were acquired from each sample. The Raman intensity for each film thickness was calculated as the average of ten randomly selected measurement positions to determine the “Average Raman Intensity”. Excitation at 785 nm produced significantly enhanced Raman signals compared to the pristine S-DNTT-10 film (0 nm), attributable to increased intensity and improved signal-to-noise ratios at characteristic vibrational peaks. These findings confirm the successful implementation of SERS under the 785 nm excitation condition.

Moreover, Figure 4c demonstrates that the Raman intensity enhancement of the S-DNTT-10 film under 785 nm excitation varied with the thickness of the deposited Au thin films. The results show that the overall Raman intensity was highest at a Au thickness of 10 nm across most spectral regions. However, within the 1510–1580 cm^−1^ range, the intensities at 5 nm and 10 nm Au thickness were compared, showing no significant difference. These results are critical for SERS–based characterization of S-DNTT-10, providing valuable insight into the optimal Au film thickness required for maximizing Raman enhancement.

In relation to the observed SERS effect, this study systematically investigates its underlying mechanisms, focusing on two widely accepted theoretical models [42,43]: (i) the electromagnetic enhancement mechanism involving surface plasmon (SP) induction on metallic substrates, and (ii) the chemical enhancement mechanism, attributed to charge-transfer interactions at molecule–metal interfaces.

To investigate the geometric characteristics of the gold nanostructures responsible for the enhanced SERS effect, the experimental methodology was rigorously validated using atomic force microscopy (AFM). The nanoscale resolution of AFM enabled quantitative topographical analysis and precise dimensional characterization of the gold nanostructures.

**Figure 5:**
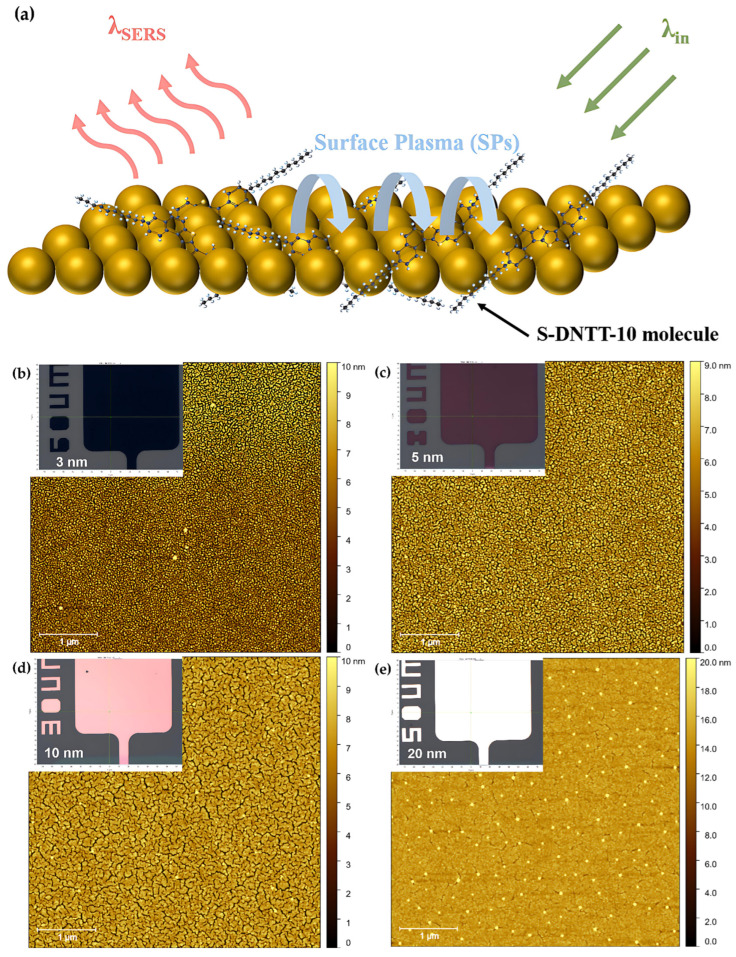

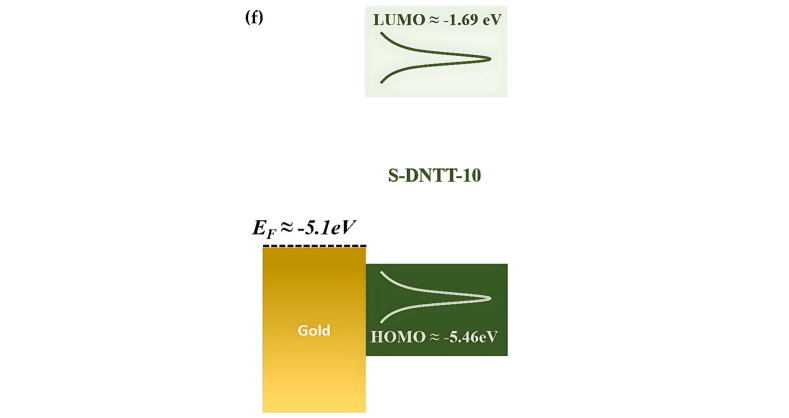
(**a**) The schematic representation of fundamental surface Plasmon (SP) mechanism of SERS. The AFM topography with top-view optical-image in different thickness of Au thin films: (**b**) 3 nm, (**c**) 5 nm, (**d**) 10 nm and (**e**) 20 nm. (**f**) The diagram of Fermi level of gold and molecular orbitals of S-DNTT-10 molecule.

Within a fixed measurement area of 5 × 5 μm^2^, the average grain sizes of Au nanostructures deposited at thicknesses of 3, 5, 10, and 20 nm were approximately 32, 41, 62, and 122 nm, respectively. The corresponding nanostructure densities were determined to be approximately 389, 238, 52, and 1.7 μm^−2^. Notably, variations in Raman intensity with differing nanostructure thicknesses were observed, suggesting a correlation between nanostructure size and the degree of induced surface plasmons (SPs). Optical images under white-light illumination (insets in Figure 5b–e) further confirmed these observations by revealing the distinct surface colorations associated with each Au thickness. Furthermore, the combined analysis with the results shown in Figure 4c demonstrated that Raman intensity varied with both the excitation wavelength and the thickness (or equivalently, average size) of the Au nanostructures. The analysis indicated that the magnitude of the SERS effect is influenced by geometric parameters of the Au nanostructures, particularly their ability to modulate SPs excitation. This conclusion is consistent with previous reports [40,41,42,43,44] and supports the interpretation that the observed SERS effect is predominantly governed by the electromagnetic (EM) enhancement mechanism.

Moreover, the potential contribution of a chemical enhancement mechanism—such as charge transfer—cannot be ruled out. This inference is supported by the energetic alignment between the Fermi level of gold (approximately −5.1 eV relative to the vacuum level) and the molecular orbital (MO) energy levels of an isolated gas-phase S-DNTT-10 molecule. Density functional theory (DFT) calculations were performed using **Gaussian 16** with the **B3LYP** functional and the **6-311G+ (d, p)** basis set. The results indicated that the LUMO and HOMO energy levels of S-DNTT-10 are approximately −1.69 eV and −5.46 eV, respectively, as illustrated in Figure 5f. These values were obtained through geometry optimization and single-point energy calculations. This alignment facilitates electron excitation in gold to the LUMO, followed by the emission of scattered light as these electrons return to the Fermi energy level.

**Figure 6:**
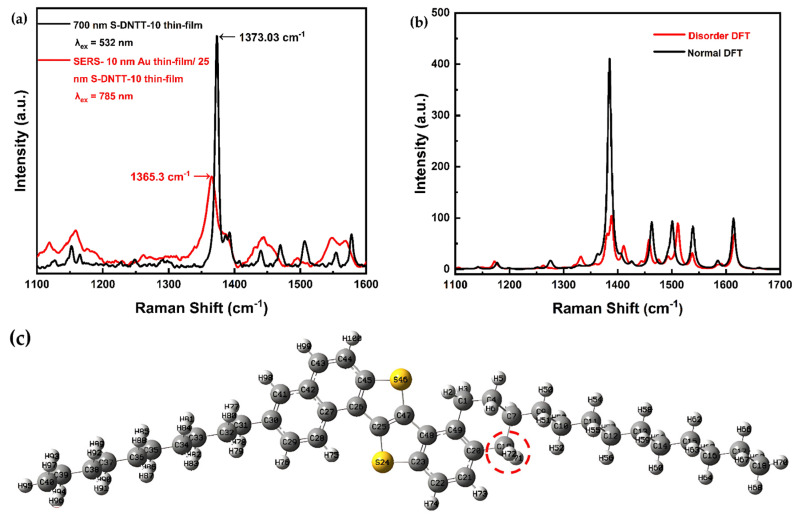
(**a**) The comparison of S-DNTT-10-SERS spectrum under 785 nm and S-DNTT-10-conventional Raman spectrum under 532 nm. (**b**) The comparison of simulated Raman spectrum of one single S-DNTT-10 molecule and hybridized S-DNTT-10 molecule. (**c**) The hypothetical S-DNTT-10 hybridized with marked hydrogen molecule.

While Raman scattering is generally considered to be independent of the excitation wavelength, this study identified deviations from this assumption. Specifically, comparison of the Raman spectrum of a 700 nm S-DNTT-10 thin film excited by a 532 nm laser revealed significant differences in the SERS spectrum, as shown in Figure 6a. The SERS spectrum exhibited a broader high-intensity range (1300–1420 cm^−1^) compared to the narrower band (1350–1375 cm^−1^) observed in the conventional Raman spectrum. In addition, several distinct bands in the conventional non-resonant Raman spectrum appeared to merge into a single, wider band in the SERS spectrum. For example, two peaks in the 1200–1300 cm^−1^ range of the Raman spectrum coalesced into a single broad peak in the SERS spectrum. This band broadening and merging are attributed to structural changes in S-DNTT-10 molecules induced by the deposition of the Au thin film. These findings are consistent with studies on the SERS effect in pentacene molecules using Au thin films [44,45]. Notably, a similar observation was reported in reference [45]. In that study, the authors discussed discrepancies between the experimental Raman spectra of pentacene thin films and the theoretical spectra calculated by DFT simulations for pentacene monomers around 1170 cm^−1^. The authors introduced an additional hydrogen atom (H atom) in their simulations as a simplified model to represent the Raman spectrum of pentacene with disordered arrangements in actual thin films. Moreover, the authors emphasized that the calculated Raman spectra revealed the effects of disorder on the pentacene molecules, suggesting that such disorder leads to enhanced Raman signal. Inspired by their approach, we applied the related concept of disorder characterization to our system (as shown in Figure 6c) to qualitatively explore and illustrate the possible origins of the experimentally observed spectral differences.

In this study, DFT simulations further confirmed that the addition of a hydrogen atom—highlighted by the red dashed circle—modifies the vibrational modes of the pentacene molecule. Based on these findings, we suggest that the thermal deposition of Au nanostructures may penetrate and structurally distort the S-DNTT-10 thin film, resulting in disordered arrangements of S-DNTT-10 molecules [36,45]. To support this hypothesis, we conducted similar DFT simulations by introducing an extra hydrogen atom into the thiophene ring of the S-DNTT-10 molecule, as shown in Figure 6c.

**Figure 7:**
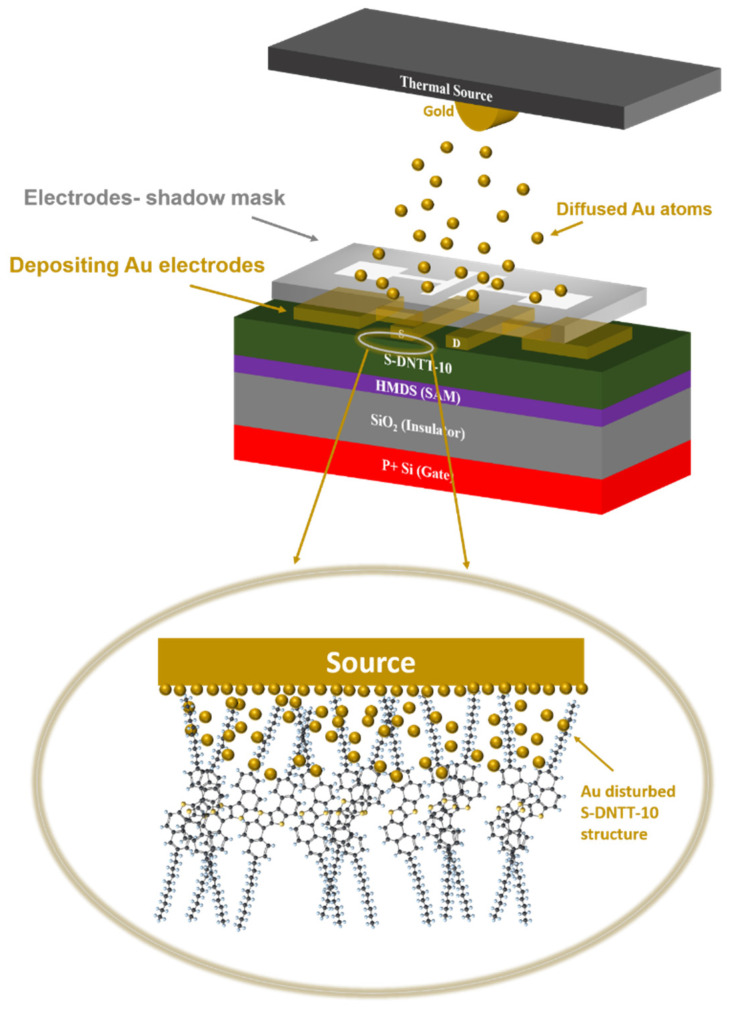
The schematic representation of Au atom potentially diffusing into S-DNTT-10 molecules.

Figure 6b compares the theoretical Raman spectra of the pristine S-DNTT-10 molecule (Normal DFT, black line) and a modified molecular structure containing an additional hydrogen atom (Disorder DFT, red line). The disorder DFT simulation reveals several notable differences, including reduced peak intensities in the 1350–1400 cm^−1^ range and peak shifts in the 1450–1530 cm^−1^ region. In particular, two distinct peaks within the 1350–1400 cm^−1^ region tended to merge into a broader spectral band. Additionally, peak shifts were observed upon comparing the SERS spectrum with that of the 700 nm thick S-DNTT-10 film. Specifically, a peak originally at approximately 1373.03 cm^−1^ in the 700 nm thick film shifted to about 1365.3 cm^−1^ in the SERS spectrum. This permanent change aligns well with similar spectral variations reported in previous SERS studies [43,44,45]. These studies also reported changes in the Raman spectrum of pentacene deposited with Au nanostructures under SERS excitation. Given that Raman scattering is directly linked to molecular polarizability changes, this explicitly links the Raman signal to molecular structural modifications. The observed vibrational modes suggest that Au nanostructures predominantly induce structural distortions in thiophene rings or alkyl chains. Accordingly, thermally deposited Au nanoparticles likely induce structural distortions in S-DNTT-10 molecules, especially near interfacial regions between the OSC thin films and electrodes, as illustrated in Figure 7, potentially affecting charge injection or extraction in devices.

The results implied that the differences observed between the SERS spectrum and the Raman spectrum of the 700 nm thick S-DNTT-10 film possibly originate from the effects of Au thin film deposition. It is hypothesized that the deposition of Au thin films modifies the molecular structure and vibrational dynamics of S-DNTT-10, leading to the observed spectral variations in Raman scattering [30]. These structural perturbations provide a plausible explanation for the observed spectral shifts, highlighting the complex interfacial interactions between metallic nanostructure deposition and molecular dynamics within organic semiconductor (OSC) films. When Au thin films are employed as electrodes in organic electronic devices, the diffusion of Au atoms and their subsequent attachment to or distortion of S-DNTT-10 molecular structures could adversely impact charge injection and extraction efficiency at the electrode/OSC interface.

Moreover, although the present study successfully investigates the SERS effects on S-DNTT-10 induced by Au nanostructures, it is important to highlight that the adsorption behavior of S-DNTT-10 molecules on Au surfaces has not yet been modeled at the DFT level. This aspect could significantly impact the vibrational properties, particularly under SERS conditions. Future investigations incorporating adsorption simulations using methods such as DMol^3^ [46], CASTEP [47], or ONETEP [48] are expected to yield a more comprehensive, molecular-level understanding of molecule–metal interactions. These extended theoretical investigations, although beyond the scope of the present work, are anticipated to complement and further deepen the insights gained in this study.

## 4. Conclusions

Our study systematically characterized the molecular vibrations of S-DNTT-10 using Raman spectroscopy to gain deeper insight into its intrinsic material properties. The non-resonant Raman spectrum of S-DNTT-10 was simulated using density functional theory (DFT), revealing characteristic vibrational modes within the 1100–1550 cm^−1^ range. Mode analysis indicated that these vibrations primarily originated from the central thiophene ring and that the combined vibrations involved the thiophene ring and alkyl chains. Particularly, the experimental non-resonant Raman spectrum measured from a 700 nm thick S-DNTT-10 film, closely aligned with the related simulated spectrum. To enhance the Raman signal for practical applications in organic electronics, Au nanostructured thin films were deposited onto S-DNTT-10 films, enabling surface-enhanced Raman scattering (SERS). Among the excitation wavelengths tested, 785 nm yielded the most effective enhancement, with Raman intensity found to be strongly dependent on Au film thickness. The optimal Au thin film thickness for SERS was determined to be approximately 10 nm. In addition, spectral shifts observed in the SERS measurements suggest that thermally evaporated Au atoms may penetrate the organic semiconductor (OSC) layer and induce local structural distortions in S-DNTT-10, particularly near electrode contact regions. Such distortions likely influence both the observed Raman features and the interfacial electronic properties of S-DNTT-10-based devices. Overall, these findings provide fundamental insights into the vibrational characteristics and interface sensitivity of S-DNTT-10, contributing to the advancement of S-DNTT-10–based organic thin-film transistors (OTFTs) and related optoelectronic applications. In particular, this technique could be employed to investigate changes in S-DNTT-10 material characteristics during charge injection or collection processes at electrode interfaces in organic electronic devices.

Although DFT simulations and Raman spectroscopy have provided valuable insights into the molecular properties of S-DNTT-10, several aspects remain to be optimized. In particular, the limited Raman signal enhancement observed from S-DNTT-10 may stem from the suboptimal configurations of thermally deposited Au nanostructures found in the geometric parameters, relative to the excitation wavelength. Consequently, weak surface plasmons (SPs) or poorly localized electromagnetic fields were generated around these nanostructures. To address this limitation, numerical methods, such as finite-difference time-domain (FDTD) simulations, should initially be employed to accurately model SP generation. However, a key challenge arises from the geometric mismatch between simulated and experimentally fabricated Au nanostructures. This discrepancy mainly stems from the use of physical vapor deposition (PVD) instead of conventional methods like chemical synthesis or self-assembly. This makes geometric uniformity difficult to achieve. Moreover, deposition onto OSC thin films with inherently irregular morphology, rather than flat substrates like glass or silicon, adds further variability. These factors contribute to deviations between simulation and experiment, undermining the reliability of the results. To mitigate these issues, the optimization of deposition parameters—such as reducing the deposition rate—could facilitate the formation of well-ordered Au nanostructures on S-DNTT-10 films, thus enhancing SP intensity at the desired excitation wavelength. Nevertheless, fabricating Au nanostructures consistent with simulation results remains challenging. Conventional techniques, including high-temperature annealing and solution-based chemical synthesis, are often incompatible with the thermal and chemical sensitivities of S-DNTT-10. The intrinsic fragility of OSC materials to external processing conditions thus imposes significant constraints on available fabrication strategies. Therefore, the development of a reliable and OSC-compatible method for fabricating well-defined Au nanostructures remains a critical challenge for future research.

## Figures and Tables

**Table 1:** The details of DFT simulated vibrational modes.

Mode	Raman Shift/cm^−1^	Vibrational Modes Descriptions
1	1141.79	C–H bond bending at alkyl chains
2	1177.11	C–H bond bending located at the aromatic rings and coupled with few slight deformations of thiophene rings
3	1251.53	C–H bond bending at alkyl chains; C–H bond bending coupled with slight breathing stretching aromatic rings and thiophene rings deformed
4	1274.84	C–H bond bending at alkyl chains; some serious C–H bond bending located at C(38), C(33), C(93), C(86), C(81) and C(45), and coupled with few slight deformation of aromatic and thiophene rings
5	1328.95	C–H bond anti-symmetry bending at alkyl chains coupled with C–C bond movement at aromatic and thiophene rings
6	1362.20	C–H bond bending at alkyl chains; deformation of thiophene rings and linked aromatic rings; seriously C–H bond bending located at C(36), C(95), C(84) and C(47); C–C bond stretched at aromatic and thiophene rings
7	1384.79	C–H bond slightly bending at alkyl chains; strongly C–H bond bending located at C(38), C(33), C(86) and C(81) coupled with deformation of aromatic and thiophene
8	1406.24	C–H bond slightly bending at alkyl chains; strongly C–H bond bending located at C(36), C(93), C(84) and C(85) and C(42)=C(90) double-bond stretching coupled with deformation of aromatic and thiophene
9	1426.09	slight C–H bond bending at the end of alkyl chains; strong C–H bond bending at C(36), C(33), C(95), C(86), C(81) and C(47) coupled with deformation of aromatic and thiophene rings.
10	1462.96	C(42)–C(43) and C(90)–C(91) strongly stretching coupled with deformation of aromatic and thiophene rings
11	1500.85	C–H slightly bending at the end of alkyl chains; deformation of aromatic and thiophene rings
12	1539.00	strongly C(42)=C(90) double-bond stretching coupled with deformation of aromatic and thiophene rings

## Data Availability

The original contributions presented in this study are included in the article. Further inquiries can be directed to the corresponding authors.

## Supplementary Materials

The following supporting information can be downloaded at: https://www.mdpi.com/article/10.3390/ma18102389/s1, Figure S1: (a) The illustration of interface of Molview website. (b) The model of S-DNTT-10 molecule was established in the interface. The left part suggested the 2D structure and the right part represented the 3D structure. Figure S2: (a) The summary of Gaussian Calculation by Lee-Yang-Parr correlation (B3LYP) functional method and the 6-311G+ (d, p) basic set. The value of imaginary frequency in the Gaussian Calculation Summary. (b) The representation of optimized molecular structure with ground state energy. (c) The partial spectrum ranging from 1100 cm^−1^ to 1700 cm^−1^. (d) The entire Raman vibrational spectrum of one single S-DNTT-10 molecule. Figure S3: The raw data of Raman spectra on S-DNTT-10/Au thin films under 785 nm excitation source. The Zip contains GIFT that simulated molecular vibration of 1100–1550 cm^−1^ in details.
