# Supplementary material for: Studies of Raman-Scattered Technology on S-Shaped Dinaphtho[2,1-b:2′,1′-f]thieno[3,2-b]thiophene-10 (S-DNTT-10)
Source: Materials (Basel). 2025 May 20;18(10):2389. doi: 10.3390/ma18102389 (PMC12113420; doi:10.3390/ma18102389)
Supplement: Supplementary file 1 [file materials-18-02389-s001.zip › materials-3582312-supplementary.pdf]

Supplementary Information

The study of Raman scattering on S-DNTT-10 thin films

Modeled the molecular structure

The molecular formula derived from Ref [1] and the structure was modeled on the website named ***Molview*** **(**https://molview.org/) as Figure S1 shown. It was a free website with easily operational interface. It stored kinds of conventional molecular information in the database such as pentacene, DNTT. It supported the structure files which can be input into relevant calculated engineering software such as ***Gaussian 09W***.

(a)

(b)

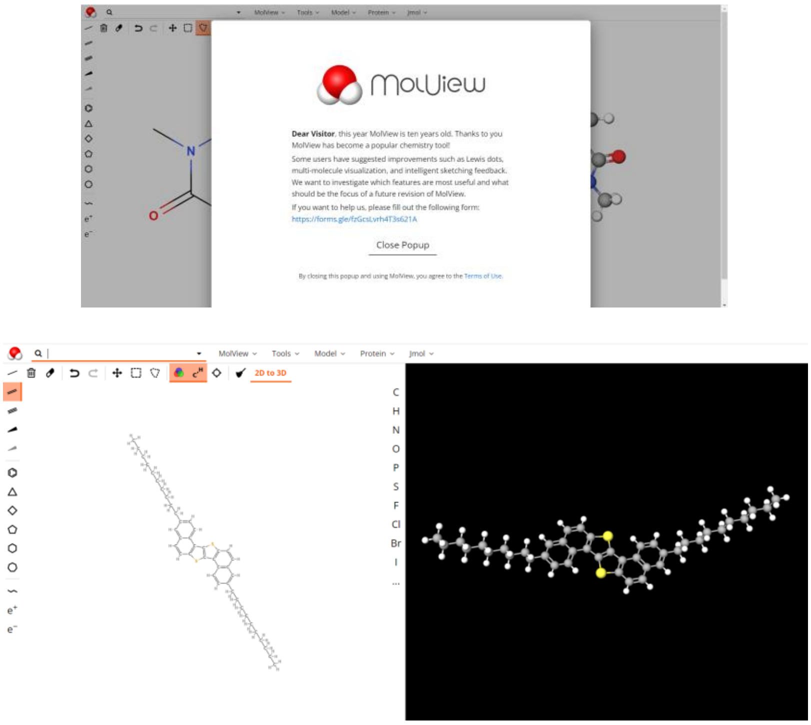

Figure S1. (**a**) The illustration of interface of *Molview* website. (**b**)The model of S-DNTT-10 molecule was established in the interface. The left part suggested the 2D structure and the right part represented the 3D structure.

DFT calculation

DFT (Density Functional Theory) was one common method of quantum chemical calculation and meanwhile it was essential to ***Gaussian*** calculated software. It can assist in obtaining the theoretical Raman spectrum and supplying the details of vibrational modes. The proved standard of results was the value of ***imaginary frequency*** was zero. And it exhibited the whole Raman vibrational spectrum of one single S-DNTT-10 molecule.

(a)

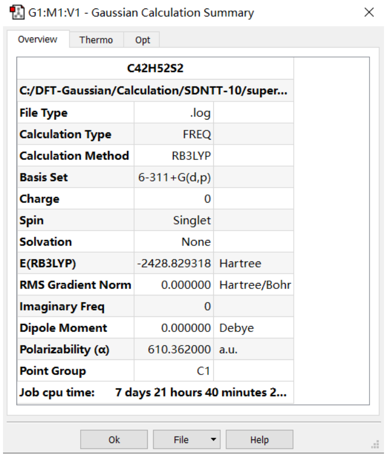

(b)

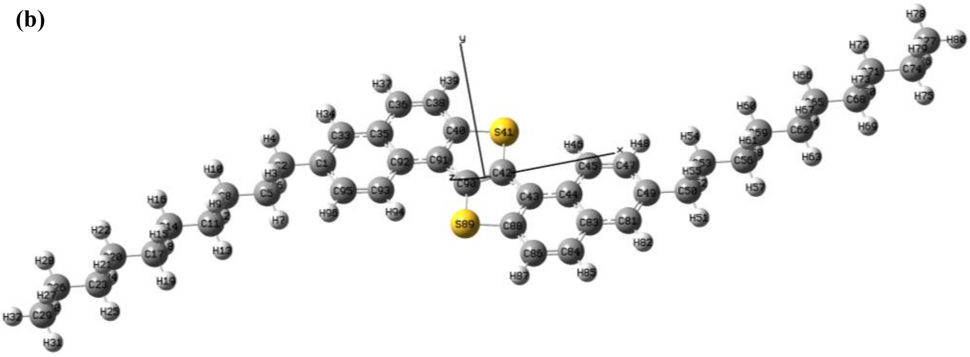

(c)

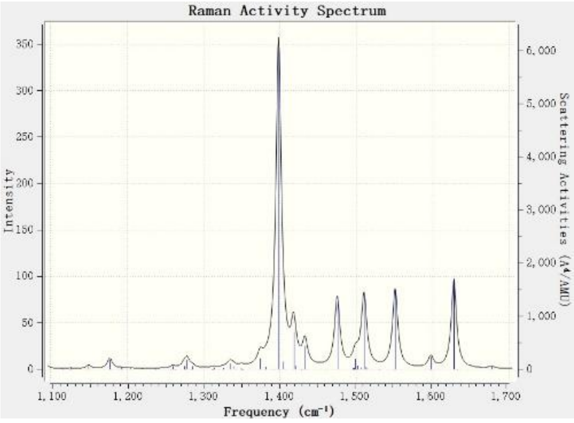

(d)

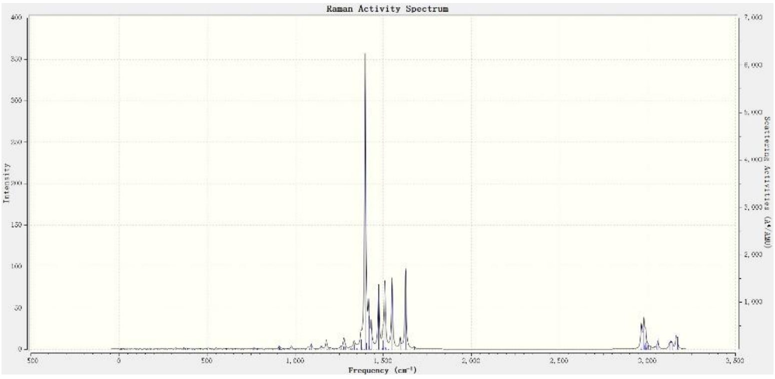

Figure S2. (**a**) The summary of Gaussian Calculation by Lee-Yang-Parr correlation (B3LYP) functional method and the 6-311G+ (d, p) basic set. The value of imaginary frequency as zero in the corresponding calculation summary. (**b**) The representation of optimized molecular structure with ground state energy. (**c**) The partial spectrum ranging from 1100 cm^{-1} to 1700 cm^{-1}. (**d**) The entire Raman vibrational spectrum of one single S-DNTT-10 molecule.

The raw data of Raman map

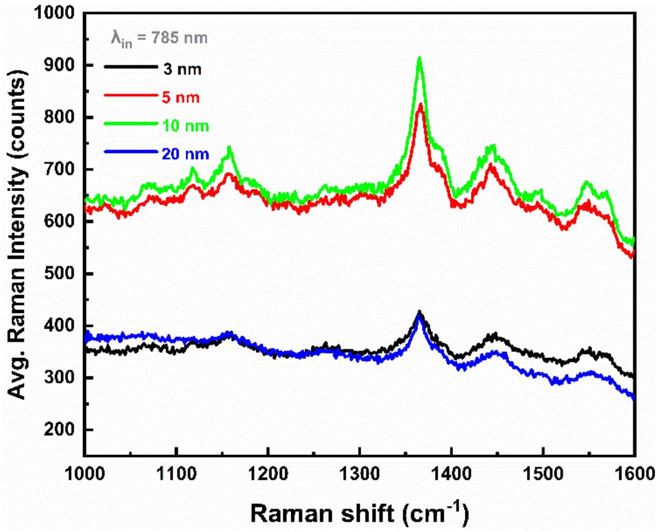

Figure S3. The raw data of Raman spectrums on S-DNTT-10/Au thin-films under 785 nm excitation source.

Reference

1. Yamaguchi, Y.; Kojiguchi, Y.; Kawata, S.; Mori, T.; Okamoto, K.; Tsutsui, M.; et al. Solution-Processable Organic Semiconductors Featuring S-Shaped Dinaphthothienothiophene (S-DNTT): Effects of Alkyl Chain Length on Self-Organization and Carrier Transport Properties. *Chem. Mater.* **2020**, *32*, 5350–5360.
